# Progress and Recent Advances in Solid Organ Transplantation

**DOI:** 10.3390/jcm11082112

**Published:** 2022-04-11

**Authors:** Charat Thongprayoon, Wisit Kaewput, Pattharawin Pattharanitima, Wisit Cheungpasitporn

**Affiliations:** 1 Division of Nephrology and Hypertension, Department of Medicine, Mayo Clinic, Rochester, MN 55905, USA; charat.thongprayoon@gmail.com; 2 Department of Military and Community Medicine, Phramongkutklao College of Medicine, Bangkok 10400, Thailand; wisitnephro@gmail.com; 3 Department of Internal Medicine, Faculty of Medicine, Thammasat University, Pathum Thani 12120, Thailand; pattharawin@hotmail.com

Over the past decade, the number of organ transplants performed worldwide has significantly increased for patients with advanced organ failure [1,2,3,4,5]. In the United States, 41,354 organ transplants were performed in 2021, increasing by 5.9% compared to 2020 [6]. While there have been significant improvements in the short-term survival of solid organ transplant recipients due to advances in immunosuppression and transplant techniques [1,2,7], long-term graft and patient outcomes still lag behind and remain areas for improvement in solid organ transplantation [2].

In this Special Issue, “Progress and Recent Advances in Solid Organ Transplantation”, researchers from different disciplines with different expertise and resources highlighted the novelty of their recent investigations in the field of organ transplantation, including issues related to donors, allografts, and patient survival [8,9,10,11,12,13,14,15,16,17,18,19,20]. While there have been significant advances in regional and national kidney paired-donation programs in matching incompatible pairs, data suggest that there may be a role for desensitization in select cases to facilitate organ transplantation [21]. In this Special Issue, Weinhard et al. summarized the roles of tocilizumab and desensitization in kidney transplant candidates [18]. In addition to progress in desensitization and preoperative monitoring of donor-specific antibodies, this Special Issue also provided insights into the monitoring and management of chronic active antibody-mediated rejection [17]. Furthermore, investigators also shed light on post-transplant complication research, including osteoporotic fractures [9], diarrhea [15], psychological changes [19], and recurrent primary disease [17].

Immunosuppression management is essential for patient and graft survival in transplant recipients [22,23,24], and studies have demonstrated the impacts of tacrolimus metabolism rates on outcomes after transplantation [25,26,27]. In this Special Issue, Kolonko et al. found the novel findings of influences of body composition parameters assessed by bioimpedance analysis on the tacrolimus metabolism, which may potentially be useful in optimizing initial tacrolimus dosing [10]. Additionally, while fast tacrolimus metabolism is associated with lower renal function after kidney transplantation [26,27], in this Special Issue, Thölking et al. found no significant impact of fast tacrolimus metabolism on dyslipidemia parameters [13].

Better understanding of subgroups of transplant recipients, such as older transplant recipients and Black transplant recipients, can help the transplant community to identify individualized strategies to improve outcomes among these vulnerable populations [11,14,28]. In this Special Issue, Zompolas et al. conducted a retrospective study to evaluate outcomes of 85 kidney transplant recipients aged ≥ 75 years in the Eurotransplant Senior Program from January 2010 to July 2018 at the Charité-Universitätsmedizin Berlin in Germany [11]. The investigators demonstrated comparable outcomes among older patients compared to their younger counterparts [11], confirming excellent outcomes, including in patient and graft survival, in carefully selected older kidney transplant recipients aged ≥ 75 years [29,30,31]. Lastly, in this Special Issue, we reported outcomes of kidney transplant recipients with sickle cell disease (SCD) from an analysis of the 2000–2019 United Network for Organ Sharing (UNOS)/Organ Procurement and Transplantation Network Database [14]. In this study, we found that SCD was significantly associated with lower patient survival and death-censored graft survival compared to non-SCD recipients. The findings of our study suggest that urgent future studies are required to identify strategies to improve outcomes in SCD kidney recipients. Additionally, the assignment of risk adjustment for SCD patients should be considered.

In summary, the findings published in this Special Issue provide novelty and additional knowledge and may help the transplant community to ultimately improve the management and outcomes of patients with solid organ transplantation.
